# Amyloid-β “Co-assembles” with Coatomer Subunit Delta (δ-COP)

**DOI:** 10.1021/acs.jpclett.5c03565

**Published:** 2026-03-19

**Authors:** Anastasia Vlachou, Om Shanker Tiwari, Ehud Gazit, Phanourios Tamamis

**Affiliations:** † Artie McFerrin Department of Chemical Engineering, 14736 Texas A&M University, College Station, Texas 77843-3122, United States; ‡ The Shmunis School of Biomedicine and Cancer Research, George S. Wise Faculty of Life Sciences, 26745 Tel Aviv University, Tel Aviv 6997801, Israel; § Department of Materials Science and Engineering, Iby and Aladar Fleischman Faculty of Engineering, Tel Aviv University, Tel Aviv 6997801, Israel; ∥ Sagol School of Neuroscience, Tel Aviv University, Tel Aviv 6997801, Israel; ⊥ Department of Materials Science and Engineering, Texas A&M University, College Station, Texas 77843-3003, United States

## Abstract

Previous studies showed that δ-COP interacts with APP and regulates its intracellular trafficking, while an important reduction in the level of Aβ plaques was observed in AD/δ-COP I422T mice. Here, we show that δ-COP interacts directly with Aβ assemblies according to experiments and simulations. Experiments suggest a two-binding site model, one with high affinity and one with lower affinity. Simulations comply with experiments and provide mechanistic biophysical insights into the high-affinity interactions, comprising a “co-assembly-like” β-sheet interaction within nearly identical domains _426_DGEYRHDS_433_ of δ-COP and _1_DAEFRHDS_8_ of Aβ, complemented by interactions between _416_GVGAPVIGEI_425_ of δ-COP and _13_HHQKLVFFAED_23_ of Aβ, with a β-bridge between δ-COP I422 and Aβ D23. As such, our simulations highlight the role of I422, which is also investigated in comparison to T422. Our studies can provide impetus for the future investigation of the interaction between δ-COP and Aβ, particularly in its involvement in intracellular trafficking in Alzheimer’s disease.

## Graphical abstract

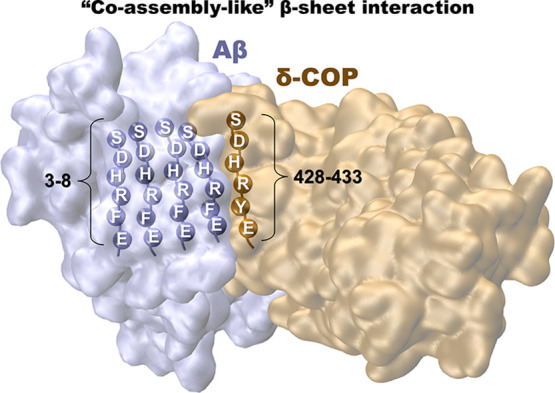

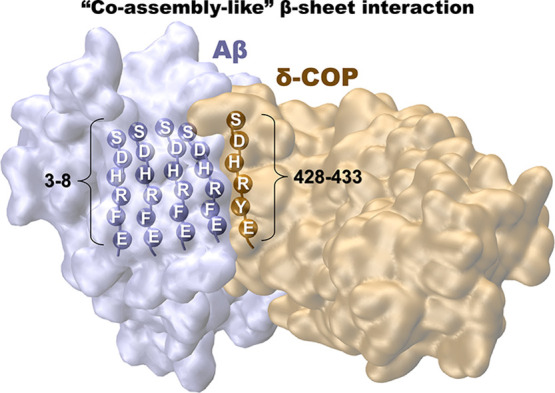

COPI subunit δ (δ-COP) interacts with amyloid precursor protein (APP) and regulates APP intracellular trafficking rather selectively, controlling its maturation and thus Aβ polypeptide production. In an *in vivo* AD model crossed with Nur17 mice (I422T, termed δ-COP mut), the reduction of the δ-COP function rescued AD pathology by significantly decreasing the amyloid plaque load and improving memory impairment.(3) Nevertheless, the exact link between Aβ and δ-COP is not well understood. It is also unclear how the Ile422Thr mutation in only 17 mice in an *in vivo* AD model leads to a reduction of δ-COP function.

Intracellular Aβ oligomers likely contribute to the early synaptic pathology in AD(3). There is a bidirectional transport between the endoplasmic reticulum (ER) and the Golgi mediated by COPI (including δ-COP) and COPII carriers, which is a highly dynamic process.(4) Aβ oligomers, considered as the toxic form of Aβ, can disrupt ER function, leading to ER stress. However, several aspects remain unclear, including the trafficking of Aβ from the Golgi apparatus to the ER.

The δ-COP domain of residues 414–435 is highly important for its functionality.(7) This domain of δ-COP comprises I422 and _426_DGEYRHDS_433_, which are both exposed to the surface. _426_DGEYRHDS_433_ of δ-COP is highly sequentially similar to _1_DAEFRHDS_8_ of Aβ, and _672_DAEFRHDS_679_ of APP. This _1_DAEFRHDS_8_ domain is expected to be flexible and exposed in Aβ assemblies, is unresolved in several PDB entries, and serves as an epitope for aducanumab and lecanemab.(8) Additionally, Aβ cross-interacts with homologous proteins, including IAPP forming an amyloid steric zipper nearly throughout the structure driven by the sequence similarity and alike physicochemical properties between the two.(9) Considering the above, the fact that proteins can self-assemble with their identical domains forming in-register parallel β-sheets, and the high degree of structural similarity between Aβ_3–8_ and δ-COP_428–433_ (RMSD = 0.71 Å considering the structural conformations used in this study and provided below), we considered investigating the possibility of interaction between Aβ and δ-COP to be highly important, such that their corresponding Aβ_3–8_ and δ-COP_428–433_ regions can co-assemble.

Our studies started by modeling δ-COP (AF-P48444-F1-v4),(10) truncated to its functional MHD domain of residues 271–511, in complex with a short Αβ peptide fibril (comprising five monomers) from meningeal AD brain tissue.(11) The modeling was performed by superimposing the backbone atoms of the two sequential similar domains of δ-COP and the external Aβ monomer, with the former comprising a β-sheet strand reminiscent of an extension of the Aβ assemblies. Upon modeling, in addition to the interface between δ-COP (_428_EYRHDS_433_) and Αβ (_3_EFRHDS_8_), we observed the appearance of another interface formed spanning from _416_GVGAPVIGEI_425_ of δ-COP and _13_HHQKLVFFAED_23_ of the first most proximal Αβ monomer (Aβ_1_) (Figure 1A).

**Figure 1:**
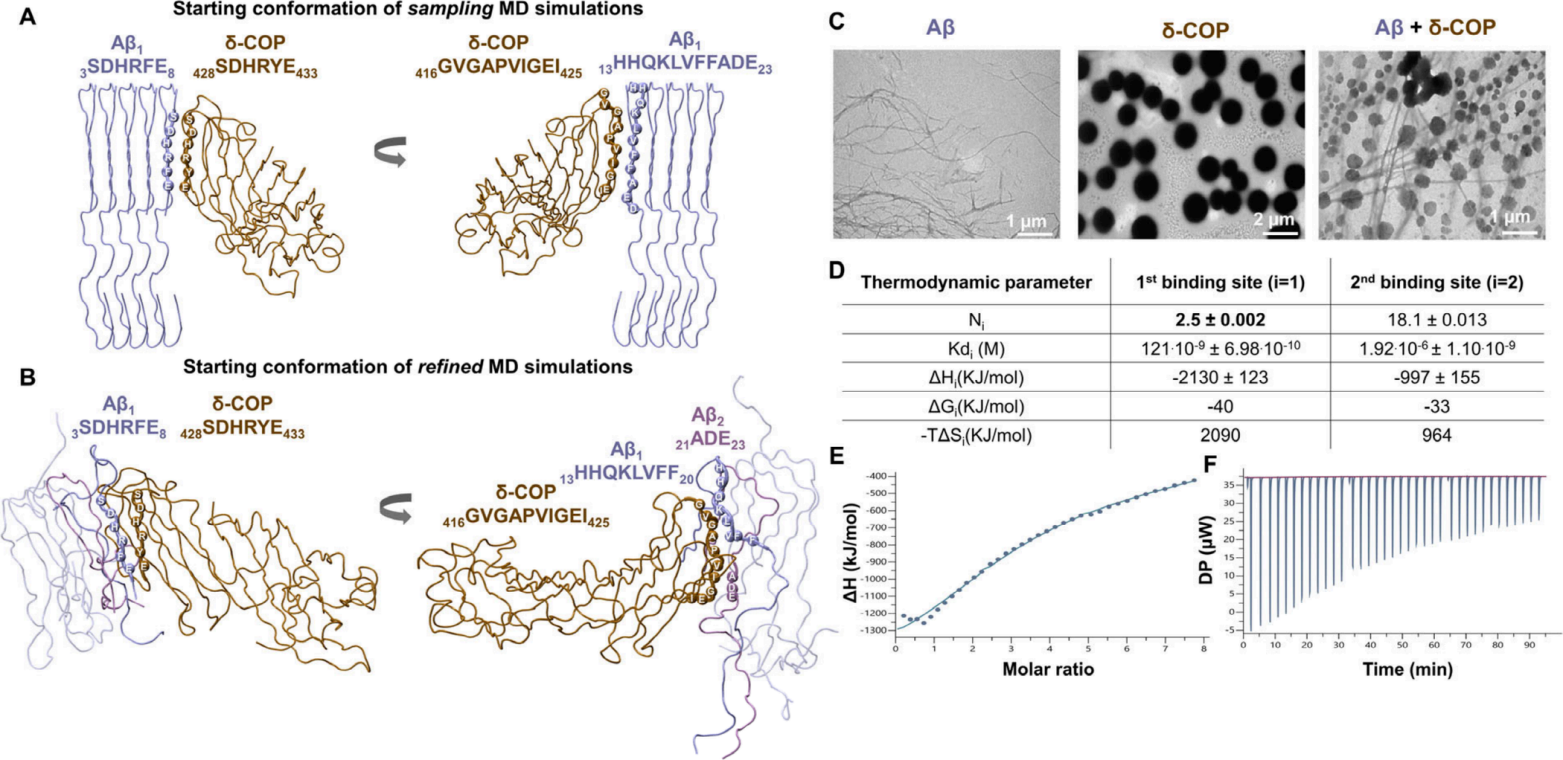
Molecular graphics of (A) δ-COP (residues 271-511) in complex with Αβ assemblies upon backbone atom superposition and (B) the lowest-binding free energy δ-COP (residues 271-511) model in complex with Αβ assemblies before the refined simulations. Molecular graphics were generated in VMD,(25) showing δ-COP in ochre and Aβ assemblies in ice-blue tubes, with residues of interest shown in VdW representation of their C atoms. (C) TEM images of Aβ fibrils, δ-COP, and a 1:1 mixture of Aβ and δ-COP, each at 10 μM in 1× PBS (pH 7.4). (D) Thermodynamic parameters generated from ITC. (E) Heat flow profile recorded during the titration of Aβ fibrils into a δ-COP solution. (F) Enthalpy changes plotted vs the molar fraction of Aβ fibrils.

At first, we used molecular dynamics (MD) simulations to sample the initially modeled binding conformations of δ-COP in complex with Aβ assemblies. In five of eight runs, δ-COP remained strongly bound to the Aβ assemblies, maintaining the “co-assembly-like” β-sheets, while in all runs, at least one residue pair contact between _416_GVGAPVIGEI_425_ of δ-COP and _13_HHQKLVFFAED_23_ remained throughout the simulations (Figure S1). Subsequently, the lowest-binding free energy snapshots from the two trajectories with the lowest average binding free energy according to PRODIGY were extracted and used as starting conformations in a second round of two sets of triplicate MD simulations (Figure S1). Due to the absence of an initial model, and given our hypothetical initial model based on the “co-assembly-like” interaction, the sampling stage was critical to provide plausible models of δ-COP in complex with Aβ assemblies.

The two lowest-binding free energy conformations, from the sampling runs, were further investigated using additional triplicate simulations (e.g., 1 μs per replicate, 6 μs total aggregated time), providing additional refinement to the system, allowing us to examine in detail the formed interactions, and providing mechanistic insights into complex formation. Among the two, the one with the lowest average binding free energy across its triplicate runs (−9.40 ± 0.37 kcal/mol) was identified using PRODIGY (Figure S2). Notably, while the simulated models share some similarities, we focused on the one with the lowest average binding free energy (Figure 1B). Interestingly, an additional β-bridge was formed between I422 of δ-COP and residue D23 of the second most proximal Aβ monomer (Aβ_2_); this was initially present and was also adequately maintained within its triplicate runs. It is also important to note that the same β-bridge was formed between I422 of δ-COP and residue D23 of the first most proximal Aβ monomer (Aβ_1_) in the other case, corresponding to the second lowest average binding free energy.

We used experiments to investigate the interaction between Aβ fibrils and the entire δ-COP in vitro using transmission electron microscopy (TEM). The TEM image qualitatively demonstrates the interaction between the Aβ fibrils and δ-COP, which leads to the conversion of smaller fibers to elongated fibrils (Figure 1C). Isothermal titration calorimetry (ITC) (Figure 1D–F and Figure S3) suggests a two-binding site model, as analysis shows a notable lower error level when the two-binding site model was applied. The *K*_d_ values measured were 121 nM (Δ*G* = −9.44 kcal/mol) with a stoichiometry of ∼2.5 and 1.92 μM (Δ*G* = −7.80 kcal/mol) with a stoichiometry of ∼18. The latter, lower affinity binding, which is ∼16 times weaker than the former, likely corresponds to less specific binding between multiple monomers in the assembly with δ-COP. On the contrary, the former, higher-affinity binding, corresponds to specific binding, of which its stoichiometry could be potentially explained by our simulated model, according to which two Aβ monomers interact with one δ-COP protein. This can be further supported by the fact that its corresponding binding free energy of −9.44 kcal/mol is in exceptional agreement with the average binding free energy of the triplicate simulations, equal to −9.40 ± 0.37 kcal/mol (Figure S2). Our results suggest that the high-affinity binding between δ-COP and Aβ assemblies can be mechanistically attributed to interactions between δ-COP with two Αβ monomers: the first most proximal Aβ monomer to δ-COP (Αβ_1_) and the second most proximal Aβ monomer to δ-COP (Αβ_2_) (Figure 1B).

Biophysical analysis and particularly the analysis of interactions between the residues of δ-COP and the first two most proximal Aβ monomers to δ-COP across triplicates indicated the preservation and reproducibility of interactions with respect to the starting conformation (Figures S4–S6) and highlighted the presence of four binding interfaces (BIs) between δ-COP and the first two Αβ monomers within assemblies (Figure 2A,B).

**Figure 2:**
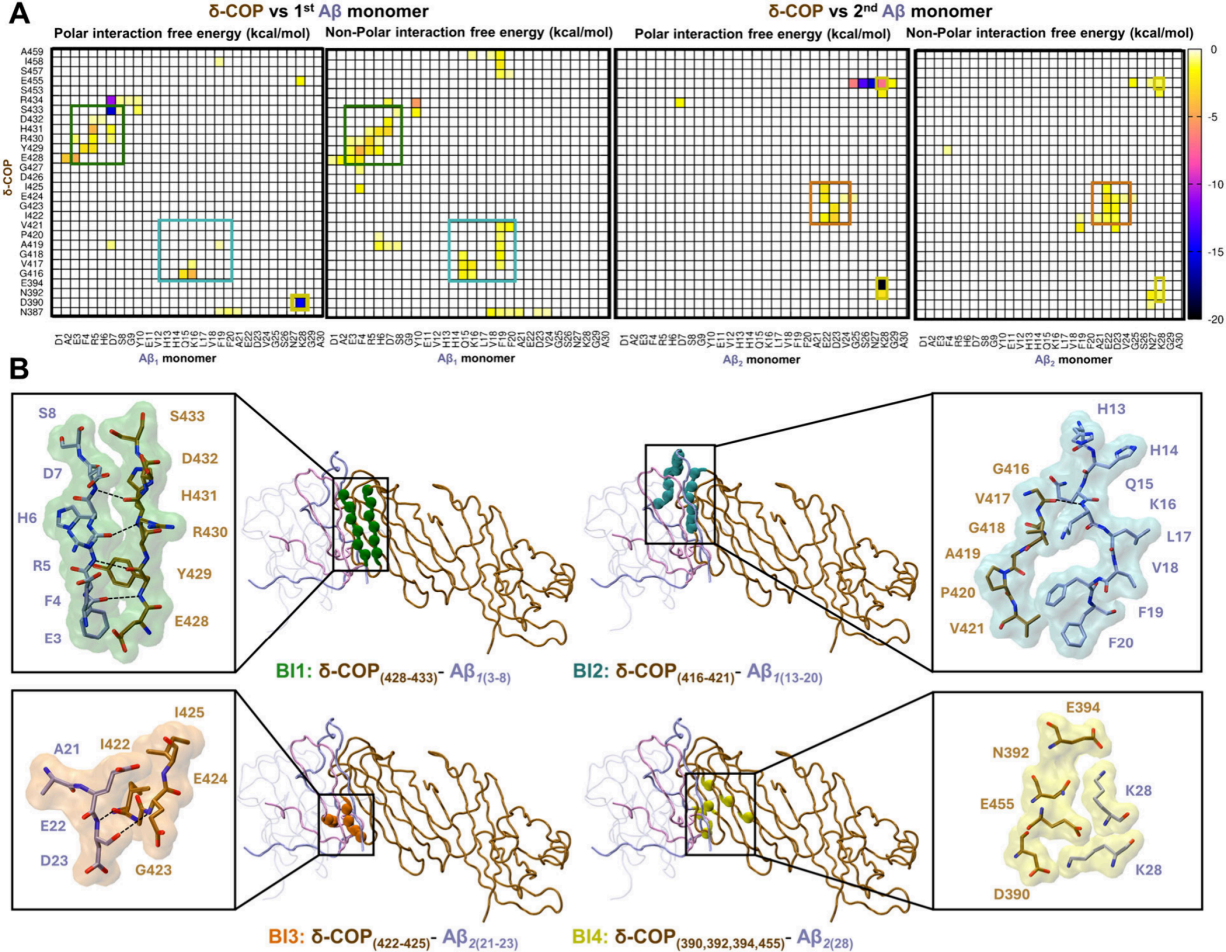
(A) Maps showing time-average polar and non-polar residue pairwise interaction free energies between residue pairs of δ-COP and the first Aβ monomer or second Aβ monomer within the lowest-average binding free energy replicate run of the refined simulations. The binding interfaces are highlighted in green for BI1, cyan for BI2, orange for BI3, and yellow for BI4. (B) Molecular graphics of the binding interfaces between δ-COP (ochre) and Aβ (ice-blue). The structure was extracted from the replicate with the lowest average binding free energy.

Interactions between δ-COP and Aβ_1_ include two binding interfaces: BI1 and BI2. BI1 comprises a “co-assembly-like” β-sheet interaction stabilized by β-bridges primarily among residue pairs _429_YRH_431_ of δ-COP and _4_FRH_6_ of Aβ_1_. The β-sheet hydrogen bonding in conjunction with side-chain complementarity observed in the “co-assembly-like” β-sheets region contributed to an amyloid-like steric zipper, characterized by strong polar and non-polar interactions stabilizing the interaction interface between δ-COP and Αβ assemblies. In particular cases, sodium and chloride ions are also in the proximity of negatively charged and positively charged amino acids in BI1, contributing to the stabilization (Figure S7). BI2 comprises interactions between _416_GVGAPV_421_ of δ-COP and _13_HHQKLVFF_20_ Αβ_1_. Interactions in BI2 involve a hydrogen bond between G416_(δ-COP)_ with K16_(Αβ1)_ or H13_(Αβ1)_, along with hydrophobic interactions between A419_(δ-COP)_ with the non-polar part of K16_(Αβ1)_ and V421_(δ-COP)_ with _19_FF_20(Αβ1)_. Synergism between BI1 and BI2 also contributes to additional non-polar interactions between H6_(Αβ1)_ and A419_(δ-COP)_ (Figure 2A). Interactions between δ-COP with Aβ_2_ also include two binding interfaces: BI3 and BI4. BI3 comprises interactions between _421_VIGEI_425_ of δ-COP and _21_AED_23_ of Αβ_2_, including a β-bridge between I422_(δ-COP)_ and D23_(Αβ2)_, a hydrogen bond between E424_(δ-COP)_ and D23_(Αβ2)_, and one between I425_(δ-COP)_ and E22_(Αβ2)_ (Figure 2B). BI4 comprises salt bridges or strong polar interactions between K28_(Αβ2)_ and N392_(δ-COP)_, E394_(δ-COP)_, and E455_(δ-COP)_ (Figure 2B). These are stabilized additionally by a salt bridge involving Aβ_1_, K28_(Αβ1)_, with D390_(δ-COP)_ and seldom with E455_(δ-COP)_ (Figure 2B).

All aforementioned binding interfaces (BI1–BI4) are also present in the initial conformation that was extracted from the sampling runs and were maintained and further refined in the triplicate runs (Figures S8 and S9). The degree of strength and preservation of such interactions is provided in detailed maps showing the average and standard deviation values of all pairs of interactions, as well as by time series flowcharts (Figures S4, S5, and S8). Overall standard deviation values of residue-pairwise interaction free energies are low with the exception of particular interactions in BI4; this is not surprising as these comprise side-chain to side-chain interactions (Figures S4 and S5). Additionally, RMSD calculations on δ-COP with the corresponding binding residues in the assemblies depict the presence of some reasonable flexibility with respect to the initial structure due to refinement, which is not unexpected due to the intrinsically disordered nature of Aβ, and overall high degree of preservation due to refinement, when comparing with respect to the average conformation (Figure S10).

Interactions at BI3 involve a β-bridge with I422, which comprises a mutation (Ι422T in nur17 mice) in the δ-COP mut. To investigate the effect of this mutation, we performed two sets of simulations of δ-COP mut. The first set of simulations comprised eight sampling runs, reminiscent of the sampling runs performed for δ-COP (in the absence of any mutation). In these simulations, the starting conformation was the same as the one that we initially used to sample δ-COP in complex with Αβ assemblies, but in this case, I422 was mutated to T422. Among eight runs, only BI1 and BI2 were maintained in a single run, while binding interfaces BI3 and BI4 were neither formed nor maintained in any of the sampling simulations involving δ-COP mut; notably, BI3 is associated with the I422T mutation (Figure S11). While only BI1 and BI2 were formed in one only sampling run of δ-COP mut, importantly and conversely, BI1 and BI2 were formed, and BI3 was at least partially maintained or fully maintained in five or three sampling runs, respectively, of δ-COP (in the absence of any mutation) (Figures S1 and S11). It is important to note that for the sake of fairness BI3 was signified as a β-bridge of residue 422 with residue D23 of either Αβ_2_ or Αβ_1_. Comparison of the lowest-binding free energy sampling runs of δ-COP and δ-COP mut indicated that the former is more energetically favorable than the latter (Figures S1 and S11). Thus, we consider that it is highly possible that δ-COP mut may not be able to form and sustain the critical and high-affinity interactions beyond the “co-assembly-like” β-sheet region. In the second set of simulations, the starting conformation corresponded to the one that δ-COP simulated in complex with Αβ assemblies in the refined simulations, yet in this case with T422. This initial structure was used as in none of the sampling runs of δ-COP mut all binding interfaces were formed and maintained. Therefore, we considered this as a “thought” study investigating δ-COP mut binding properties to Αβ assemblies under the hypothesis that such interactions can be formed, and Αβ assemblies’ binding interface conformation can also be altered, as is the case for δ-COP, to better accommodate favorable interactions with δ-COP mut, as well. Interestingly, within these simulations, all binding interfaces were maintained, and while the average binding free energy is nearly on par with that of δ-COP (Figure S12). We suggest that this indicates two important interrelated aspects that warrant further future investigation. The I422T mutant could presumably not favor direct binding of (at least) ordered Aβ assemblies leading to the formation of a β-bridge to D23_(Aβ1)_, or D23_(Aβ2)_, perhaps consequently, due to its inability to induce a conformational change to the assemblies, leading to optimal interactions with them. In this context, our results suggest that binding of δ-COP mut to Aβ assemblies could be less (or significantly less) possible, as it may depend on the capacity of Aβ assemblies to adjust on their own into a conformation that results in favorable binding δ-COP mut; as mentioned above, such a conformation was observed in our case only when sampling δ-COP but not δ-COP mut. Altogether, these highlight the importance of our sampling simulations that were implemented to initially model the complexes. We consider that this can provide impetus for the future investigation of δ-COP mut with Αβ, and in general a variety of combinations between δ-COP mutants and Αβ disease relevant variants and polymorphs.

Interestingly, despite the fact that initially our sampling simulations started from modeling an Αβ fibril relevant to AD, upon their completion and within the refined simulations, the conformation of Aβ assemblies could be considered as a parallel ordered oligomer with weakened interactions and lower order compared to the fibril (Figure S13). This can be at least partly attributed to its interaction with δ-COP and the fact that a small number of monomers were included. As such, our simulations can be indicative of how δ-COP interacts with an Αβ oligomer. Experiments investigating fibrils instead of oligomers ensured a parallel arrangement of peptides that is typical among an abundance of Αβ fibrils resolved,(16) in line with the parallel arrangement of assemblies in the simulations. Given that our simulations show that interaction occurs primarily with the outermost monomer and primarily its less structured N-terminal domain, binding of δ-COP to less ordered oligomers or even oligomers comprising antiparallel arrangement, likely with a lower affinity, is also a possibility. Notably, our study suggests the potential critical role of some order in the Αβ oligomers for high-affinity interactions between the two. Also, δ-COP may potentially interact with segment _672_DAEFRHDS_679_ of APP, perhaps with lower affinity due to the likely lower probability of the supplementary interface to be formed; this warrants further investigation. Additionally, the diversity of oligomers formed by Aβ40 and Aβ42 could play a key role in the binding to δ-COP.(17)

Our computational structural model, presented above and represented by its triplicate runs, does not provide information about the second binding site of less specific and lower-affinity binding between δ-COP and multimers of Αβ, according to experiments. We hypothesized that this could be an outcome of the fact that we considered a truncated δ-COP protein, excluding residues 1–270, which comprises the structured domain of residues 1–134, and thus, we potentially excluded additional interactions that can occur. Upon superposition of our modeled structure (within the region of residues 380–460), modeling and simulations using a similar approach to those of the refined runs, but now including the entire δ-COP protein, we observed that the δ-COP region of residues 24–40 interacted with all amyloid monomers in the simulated model, in two out of three runs (Figure S14). We consider that this could further stabilize the interaction between δ-COP and Aβ assemblies and that the two binding sites could mechanistically correlate with the experiments suggesting a two-binding site model. Notably, while δ-COP binding to APP has been reported in the past,(1) this study presents the first, to the best of our knowledge, evidence of and mechanistic insights into δ-COP’s interaction with Αβ assemblies, also in comparison to I422T.

Modifications in the ER have been documented in AD and can cause ER stress, which has been associated with Aβ formation and accumulation.(6) ER stress plays a significant role in Aβ-induced apoptotic cell death in brain endothelial cells.(18) The disorganization of both ER and Golgi apparatus organelles exposed to Aβ oligomers has been suggested to comprise an early pathological alteration in AD.(6) Our study could pave the way for the future investigation of the potential role of the interaction of δ-COP with Aβ in AD. It would be important to study whether the accumulation of Aβ to the ER could be attributed, at least partly, to its direct interaction with δ-COP, and δ-COP’s possible role in functioning as a carrier of Aβ ordered oligomers from the Golgi apparatus to the ER. Concurrently, it would be critical to investigate whether such interaction between δ-COP and Aβ oligomers could potentially affect the trafficking and processing of other proteins from the Golgi apparatus to the ER; this could in turn negatively impact several other key cellular processes associated with δ-COP’s trafficking.

Additionally, our study provides in-depth biophysical insights into a “co-assembly-like” β-sheet interaction between a protein and an assembly, as well as a paradigm for its investigation, which would be hard to study solely using experiments due to challenges in elucidating the structure of cross-interactions, such as heteromeric interactions(19) or interactions between fibrils and other proteins. This can pave the way for understanding co-assembly-like interactions in biology and disease and providing inspiration for designing protein and peptide-assembling systems (e.g., peptide drug nanocarriers comprising co-assembling peptides with chemokine receptor targeting properties). Additionally, while much effort has been placed on Αβ inhibition, our study can provide impetus for the potential inhibition of the interaction between Αβ with δ-COP, including the design of high-affinity ligands targeting the active site of δ-COP to block this interaction.

## Supplementary Material







## Supporting Information

Detailed computational and experimental procedures and supplementary figures supporting the key findings (PDF)
Snapshots extracted every 100 ns from refined simulations of one δ-COP (271-511) replicate in complex with Αβ assemblies, with all frames aligned to the backbone atoms of the first frame (PDB)
Final simulation structure of the entire δ-COP in complex with Αβ assemblies extracted at the end of one of the replicate runs (100 ns) (PDB)
