# Supplementary material for: Amyloid-β “Co-assembles” with Coatomer Subunit Delta (δ-COP)
Source: J Phys Chem Lett. 2026 Mar 19;17(13):3783–8. doi: 10.1021/acs.jpclett.5c03565 (PMC13051450; doi:10.1021/acs.jpclett.5c03565)
Supplement: Supplementary file 1 [file jz5c03565_si_001.pdf]

Supporting Information

Amyloid-β "Co-assembles" with Coatomer Subunit Delta (δ-COP)

Anastasia Vlachou[1#], Om Shanker Tiwari[2,3,4,#], Ehud Gazit[2,3,4*], Phanourios Tamamis[1,5*]

[1]Artie McFerrin Department of Chemical Engineering, Texas A&M University, College Station, Texas 77843-3122, United States.

[2]The Shmunis School of Biomedicine and Cancer Research, George S. Wise Faculty of Life Sciences, Tel Aviv University, Tel Aviv 6997801, Israel.

[3]Department of Materials Science and Engineering, Iby and Aladar Fleischman Faculty of Engineering, Tel Aviv University, Tel Aviv 6997801, Israel.

[4]Sagol School of Neuroscience, Tel Aviv University, Tel Aviv 6997801, Israel.

[5]Department of Materials Science and Engineering, Texas A&M University, College Station, Texas 77843-3003, United States.

# Equally contributing first authors

Corresponding authors:

Ehud Gazit: ehudg@post.tau.ac.il

Phanourios Tamamis: tamamis@tamu.edu

Table of Contents

Supporting Methods 3

SM1. Modeling δ-COP in complex with Aβ assemblies 3

SM2. Sampling simulations of δ-COP in complex with Aβ assemblies and δ-COP mut in complex with Aβ assemblies 3

SM3. Refined simulations of δ-COP modeled complexes with Aβ assemblies 4

SM4. Biophysical analysis of δ-COP modeled complexes with Aβ assemblies 5

SM5. Experimental study of δ-COP/Aβ fibrils 6

Supporting Figures 7

References 20

Supporting Methods

SM1. Modeling δ-COP in complex with Aβ assemblies

We modeled δ-COP (corresponding to MHD domain (271-511)) in complex with Aβ assemblies using the following structures, respectively: AlphaFold:AF-P48444-F1-v4[1,2,3] and PDB:6SHS[4]. The MHD domain of δ-COP encompasses the region of interest ($_{428}$EYRHDS$_{433}$). The Aβ hexamer was extracted from PDB:6SHS and used to create the model of δ-COP in complex with Aβ assemblies.

The modeling was performed by superimposing the backbone atoms of the two sequential similar domains of δ-COP and the external Aβ monomer, with the former comprising a β-sheet strand reminiscent of an extension to the Aβ assemblies. Particularly, the two molecular structures, the MHD domain of δ-COP and the Aβ hexamer, using the RMSD Trajectory Tool provided by VMD[5], were aligned based on the sequence similarity between the backbone atoms of $_{428}$EYRHDS$_{433}$ and $_{3}$EFRHDS$_{8}$ of the external Aβ monomer. Upon alignment, the external Aβ monomer, based on which the alignment performed, was removed. This led to the final structural model of δ-COP in complex with Aβ assemblies, composed of the MHD domain of δ-COP and an Aβ pentamer.

SM2. Sampling simulations of δ-COP in complex with Aβ assemblies and δ-COP mut in complex with Aβ assemblies

The model of δ-COP in complex with Aβ assemblies was first simulated in triplicate, aiming to investigate the tendency of Aβ to remain stably bound to δ-COP. The set-up of the simulations was performed using the "Solution Builder" input generator from CHARMM-GUI[6,7,8]. The pH was selected to be 7.4. The δ-COP structure was modeled with acetylated N-termini and COO^- C-termini, while the Aβ assemblies had NH_3^+ N-termini and COO^- C-termini. The entire model of δ-COP in complex with Aβ assemblies was centered in a cubic periodic boundary condition (PBC) box, with a minimum distance of 15 Å between any atom and the box edge. The system was solvated with water molecules and potassium and chloride ions of 0.15 M concentration, representing the intracellular conditions. After the system was prepared using all steps provided by the "Solution Builder", a short equilibration NVT simulation (0.125 ns) was performed at 300 K, with a timestep of 0.001 ps, during which harmonic constraints were applied on heavy backbone atoms with a CHARMM[7] force constant of 400 kJ/mol·nm² and heavy sidechain atoms with a CHARMM[7] force constant of 40 kJ/mol·nm². This was followed by a 500 ns NPT production simulation at 300 K and 1 atm with a timestep of 0.002 ps, during which no constraints were applied. Equilibration and production simulations were performed in OpenMM[9], using the default parameters and setup provided by CHARMM-GUI[6,7,8]. Finally, Lennard-Jones interactions were scaled to zero at a distance of 12 Å, and the temperature was controlled by a Langevin thermostat at 300 K using a friction coefficient of 1 ps^{-1}. In all cases prior to the initiation of simulations, the

standard procedure provided by CHARMM-GUI was used, including energy minimization steps during PBC setup in CHARMM, as well as prior to the simulations using OpenMM.

The model of δ-COP mut in complex with Aβ assemblies was generated by introducing the I422T mutation in the model of δ-COP complex with Aβ assemblies, which was described above. The mutation and the setup of the simulations were performed using the "Solution Builder" input generator from CHARMM-GUI[6,7,8].

We performed eight runs in total per system. Three runs started from the same model produced in the previous section of δ-COP or δ-COP-mut in complex with Aβ assemblies, individually. The triplicate runs started from the same structure but with different initial velocities to ensure statistical independence. Five additional runs started from the aforementioned model with additional energy minimization steps included at the beginning. Particularly, in this case, the first, second, third, fourth and fifth structure of δ-COP or δ-COP-mut, individually, were subjected to (100 SD, 100 ABNR), (200 SD, 200 ABNR), (300 SD, 300 ABNR), (400 SD, 400 ABNR) and (400 SD, 400 ABNR) steps, respectively. The sampling round of simulations were performed for 500 ns each or until loss of interactions between δ-COP and Aβ assemblies was observed.

Upon completion of the **sampling** round of simulations, (δ-COP in complex with Aβ assemblies and δ-COP mut in complex with Aβ assemblies) we calculated the binding free energy between δ-COP and Aβ assemblies every 10 ns using PRODIGY[10,11.] All PRODIGY binding free energy calculations were performed locally and not on the server. The lowest binding free energy snapshots (430 ns, **sampling** run #1, and 240 ns, **sampling** run #3) between δ-COP and Aβ assemblies were extracted and used as initial conformations in the subsequent **refined** simulations.

SM3. Refined simulations of δ-COP modeled complexes with Aβ assemblies

The simulations were performed in the same conditions as the **sampling** runs, with the exception of the equilibration time, which was 5 ns, and the production time, which was 1 μs. As mentioned above, the set up of the simulations was performed using the "Solution Builder" input generator from CHARMM-GUI[6,7,8]. The pH was selected to be 7.4. The δ-COP structure was modeled with acetylated N-termini and COO- C-termini, while the Aβ assemblies had NH_3^+ N-termini and COO^- C-termini. Each modeled complex was centered in a cubic periodic boundary condition (PBC) box, with a minimum distance of 15 Å between any atom and the box edge. The systems were solvated with water molecules and potassium and chloride ions of 0.15 M concentration, representing the intracellular conditions. After the system were prepared using all steps provided by the "Solution Builder", a short equilibration NVT simulation (5 ns) was performed at 300 K, with a timestep of 0.001 ps, during which harmonic constraints were applied on heavy backbone atoms with a CHARMM[7] force constant of 400 kJ/mol·nm² and heavy sidechain atoms with a CHARMM[7] force constant of 40 kJ/mol·nm². This was followed by a 1 μs NPT production simulation at 300 K and 1 atm with a timestep of 0.002 ps, during which no constraints were

applied. Equilibration and production simulations were performed in OpenMM[9], using the default parameters and setup provided by CHARMM-GUI[6,7,8]. Finally, Lennard-Jones interactions were scaled to zero at a distance of 12 Å, and the temperature was controlled by a Langevin thermostat at 300 K using a friction coefficient of 1 ps^{-1}. The triplicate runs started from the same structure but with different initial velocities to ensure statistical independence. Hence, in total, the δ-COP modeled complexes with Aβ assemblies were simulated for 6 μs of aggregated time.

SM4. Biophysical analysis of δ-COP modeled complexes with Aβ assemblies

Upon completion of the simulations, all subsequent analyses were conducted at 10 ns intervals for each replicate run. While all monomers were included in the calculations, the biophysical analysis, described below, focused on the interactions between δ-COP with each one of the two neighboring Aβ monomers at the interaction interface, hereafter referred to as the first (Aβ1) and second Aβ monomer (Aβ2), respectively. For all the replicates, we calculated (a) the contact probabilities between residue pairs of δ-COP with each one of the two neighboring Aβ monomers, (b) the hydrogen bond occupancies between atom pairs of δ-COP with each one of the two neighboring Aβ monomers and (c) the residue pairwise interaction free energy between residue pairs of δ-COP with each one of the two neighboring Aβ monomers.

(a) Contact probabilities. We calculated the contact probabilities between residue pairs of δ-COP with each one of the two neighboring Aβ monomers, using in-house FORTRAN scripts. The contact probabilities were calculated as the total number of instances that two residues were in contact divided by the total number of analyzed snapshots. Two residues were considered in contact if the distance of any of their atoms (including hydrogens) was less than 3.5 Å (≥ 3.5 Å).

(b) Hydrogen bond occupancies. We calculated hydrogen bond occupancies between atom pairs of δ-COP with each one of the two neighboring Aβ monomers interface, using the VMD[5] Hydrogen Bond tool, with a cutoff of 3.5 Å and a cutoff angle of 90°. Similarly, using the same tool and cutoffs, we calculated the backbone sidechain or the sidechain-sidechain hydrogen bond occupancies.

(c) Residue pairwise interaction free energy. We calculated the average interaction free energy between residue pairs of δ-COP with each one of the two neighboring Aβ monomers, decomposed into polar and non-polar components[12,13,14,15,16,17], using CHARMM[7] and employing the GBMV[18,19] implicit solvent model method II, as provided by CHARMM-GUI[6,7,8,20] (for the setup of implicit solvent simulations).

The residue pairs shown in the maps were selected based on a contact probability greater than 25% across all replicate runs. Similarly, the hydrogen bond occupancies were calculated between all the adjacent Aβ monomers. Lastly, we calculated the binding free energy between δ-COP and Aβ assemblies within the **refined** simulations every 10 ns, using PRODIGY[10] for every replicate.

SM5. Experimental study of δ-COP/Aβ fibrils

Transmission Electron Microscopy (TEM): Aβ40, δ-COP (corresponding to the entire protein), and their mixture (Aβ40+δ-COP at a 1:1 ratio) were each dissolved in 1x PBS to a final concentration of 10 μM. The solutions were then heated at 90 °C for 3 hours, vortexed, and allowed to cool slowly overnight to promote gradual self-assembly. For imaging, 5 μL of each self-assembled sample (10 μM in 1x PBS) was drop-cast onto a 400-mesh carbon-stabilized, Formvar-coated copper grid (Ted Pella, California, USA). After allowing the samples to adhere for 5 minutes, excess liquid was gently removed using a lint-free tissue. The grids were then air-dried at room temperature. Morphological analysis was performed using a JEM-1400 transmission electron microscope (JEOL, Tokyo, Japan) operated at an accelerating voltage of 80 kV.

Isothermal Titration Calorimetry (ITC): Aβ was prepared at a concentration of 100 μM in 1x PBS (pH 7.4), while δ-COP was diluted to a working concentration of 10 μM in the same buffer. Titration experiments were conducted at 25 °C. The ITC instrument was equilibrated at 25 °C until a stable, flat baseline was achieved, with stirring maintained at 600 rpm. Before measurements, all solutions were incubated at 25 °C and degassed for 5 minutes. For the titration, 280 μL of δ-COP solution was loaded into the low-volume ITC instrument (Malvern MicroCal PEAQ-ITC), and 40 μL of the Aβ solution was loaded into the syringe. Aβ was titrated into PBS as a control employing the same protocol, and the resulting data were subtracted from the final measurements. The titration experiment was performed by 3 consecutive titrations, each of them are involved 13 injections of 3 μL each (the first injection being 0.4 μL), with a 150-second equilibration between each addition. The resulting data were united into one isotherm and fitted using Malvern MicroCal PEAQ-ITC Analysis Software, applying a "two sets of binding sites" model under an independent binding framework.

Supporting Figures

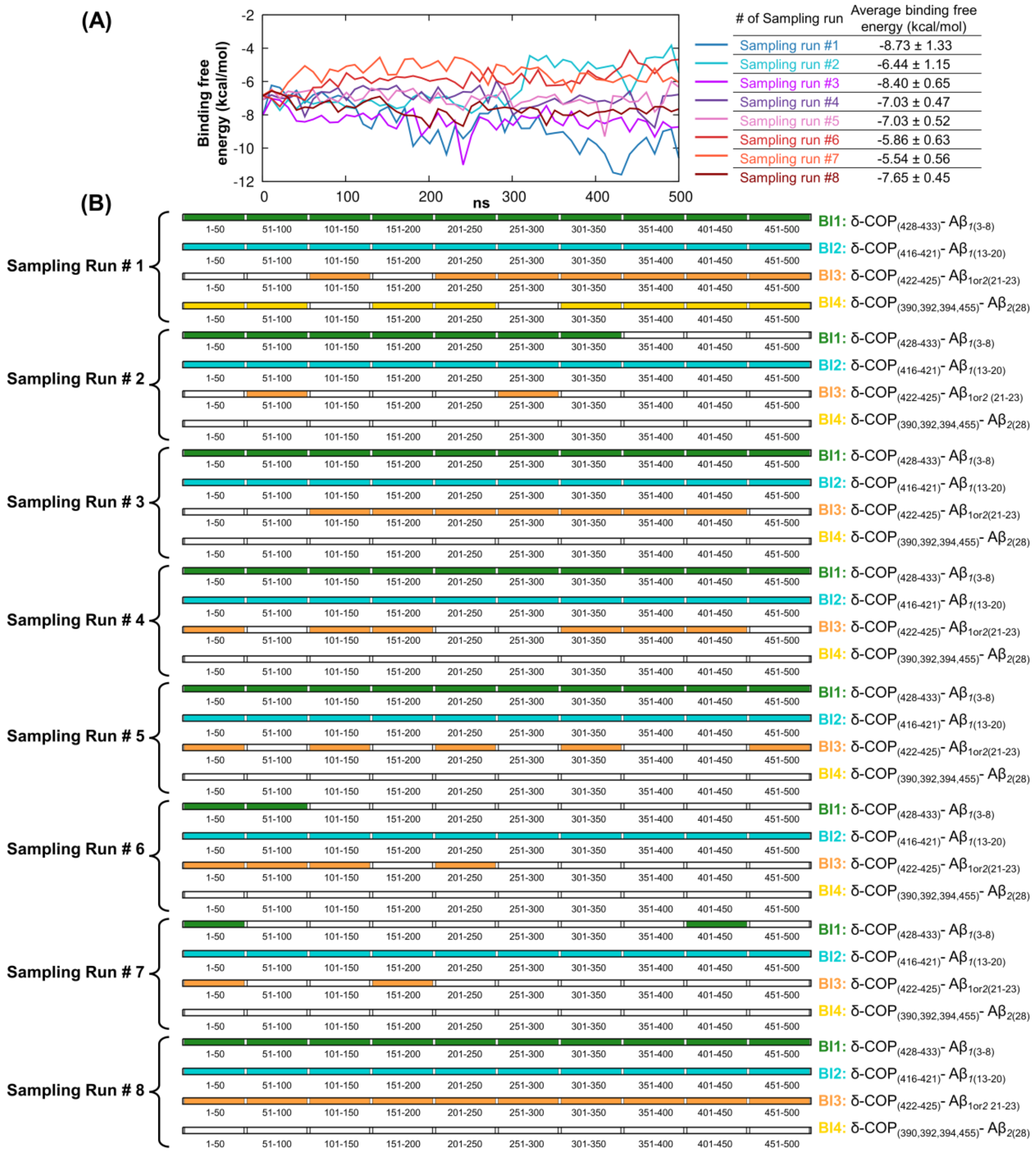

Figure S1. (A) Binding free energy (kcal/mol) of δ-COP with Aβ assemblies as a function of the simulated time (ns) for all sampling runs of δ-COP complexes with Aβ assemblies, as well as the time average values. **(B)** Binary time-window tape-diagrams showing presence (colored) or absence (uncolored) of (i) at least one β-sheet between any residues of δ-COP$_{(428\text{-}433)}$ and Aβ$_{1(3\text{-}8)}$ (green-tape-diagrams), (ii) at least one contact between any residues of δ-COP$_{(416\text{-}421)}$ and Aβ$_{1(13\text{-}20)}$ (cyan tape-diagrams), (iii) at least one β-sheet between any residues of δ-COP$_{(422\text{-}425)}$ and Aβ$_{1(21\text{-}}$

$_{23)}$ or A$\beta_{2(21\text{-}23)}$ (orange-tape-diagrams), (iv) at least one contact between any residues of δ-COP$_{(390,392,394,455)}$ and A$\beta_{2(28)}$ (yellow-tape-diagrams) for all **sampling** runs of δ-COP complex with Aβ assemblies.

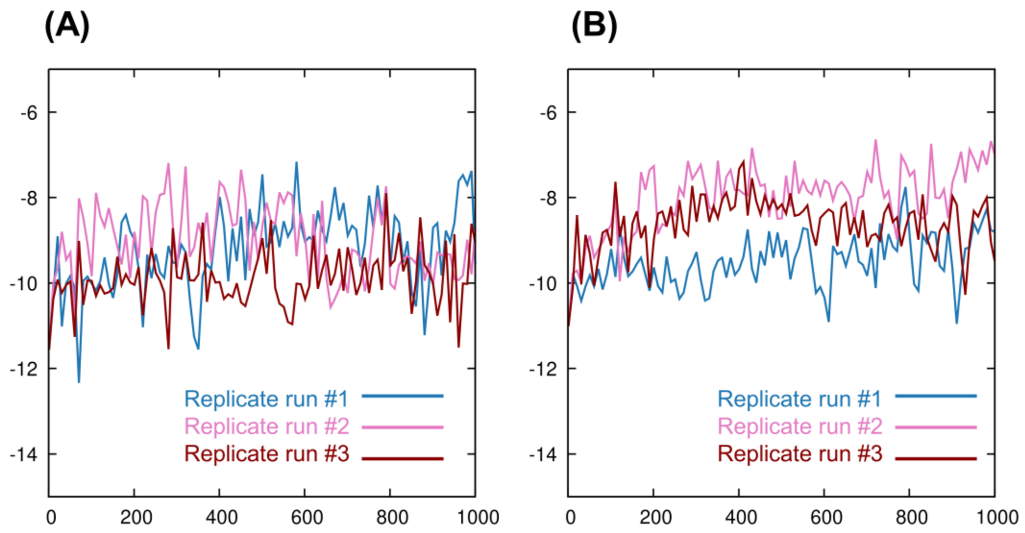

(C)

# of Replicate run	(a) Average binding free energy (kcal/mol)	(b) Average binding free energy (kcal/mol)
Replicate run #1	-9.27	-9.50
Replicate run #2	-9.03	-7.94
Replicate run #3	-9.91	-8.65
**Average**	-9.40 ± 0.37	-8.70 ± 0.64

Figure S2. (A-B) Binding free energy (kcal/mol) of δ-COP with Aβ assemblies as a function of the simulated time (ns) for all refined runs of the two lowest binding free energy starting conformations of δ-COP complexes with Aβ assemblies extracted from the sampling simulations respectively. **(C)** The time average values of the binding free energy per replicate as well as the average across the three replicates per system.

"one-binding-site" fit

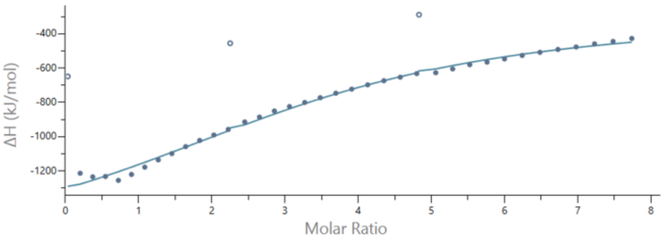

Thermodynamic parameter	1st binding site (i=1)
N_i	4.02 ± 0.258
Kd_i (M)	$11.4 \cdot 10^{-6} \pm 2.96 \cdot 10^{-6}$
ΔH_i(KJ/mol)	-1260
ΔG_i(KJ/mol)	-28.2
$-T\Delta S_i$(KJ/mol)	1230

Figure S3. ITC results upon fitting the data to the "one-binding-site" model.

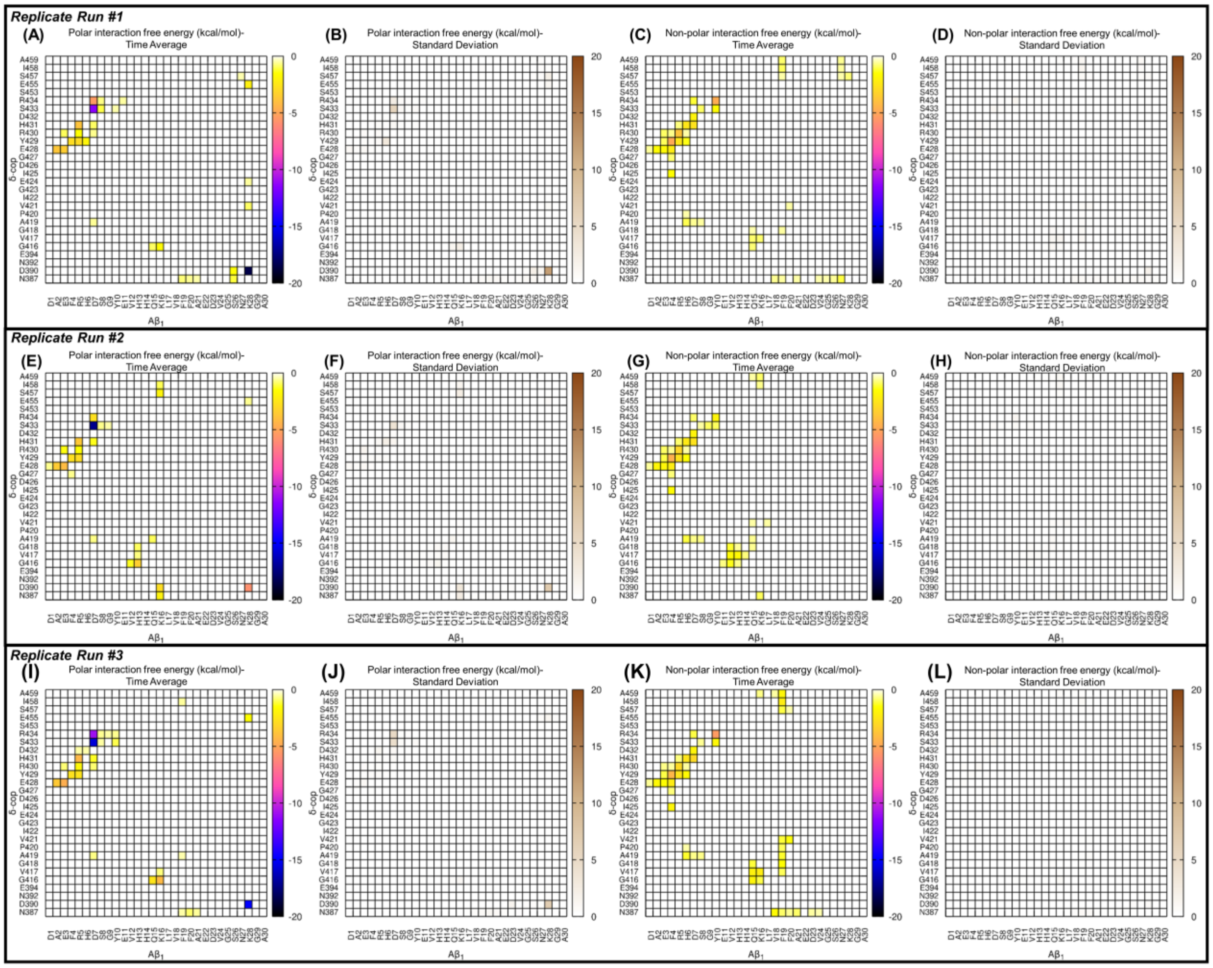

Figure S4. (A-B) Maps showing the time-average and standard deviation of polar interaction free energy between residue pairs of δ-COP/Aβ$_1$ monomer in the Replicate run #1 of the lowest binding free energy set of **refined** simulations. **(C,D)** Maps showing the time-average and standard deviation of non**-**polar interaction free energy between residue pairs of δ-COP/Aβ$_1$ monomer in the Replicate run #1 of the lowest binding free energy set of **refined** simulations. **(E-H)** Same as (A-D) for Replicate run #2. **(I-L)** Same as (A-D) for Replicate run #3. Standard deviation values of the residue-pairwise interaction free energies were calculated after averaging over 200-ns time windows.

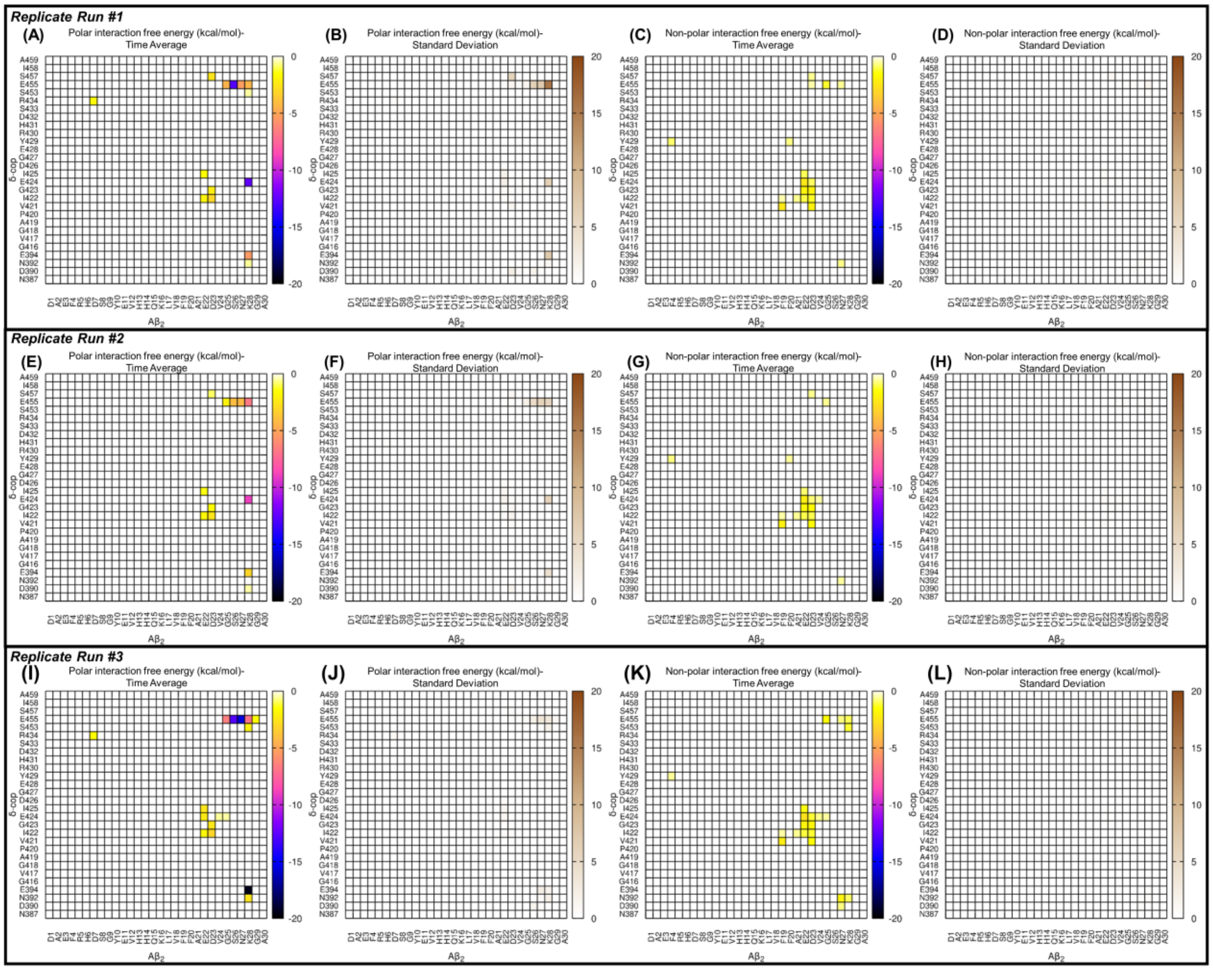

Figure S5. (A-B) Average and standard deviation of polar interaction free energy maps between residue pairs of δ-COP/Aβ_2 monomer in the Replicate run #1 of the lowest binding free energy set of **refined** simulations. **(C,D)** Average and standard deviation of non-polar interaction free energy maps between residue pairs of δ-COP/Aβ_2 monomer in the Replicate run #1 of the lowest binding free energy set of **refined** simulations. **(E-H)** Same as (A-D) for Replicate run #2. **(I-L)** Same as (A-D) for Replicate run #3. Standard deviation values of the residue-pairwise interaction free energies were calculated after averaging over 200-ns time windows.

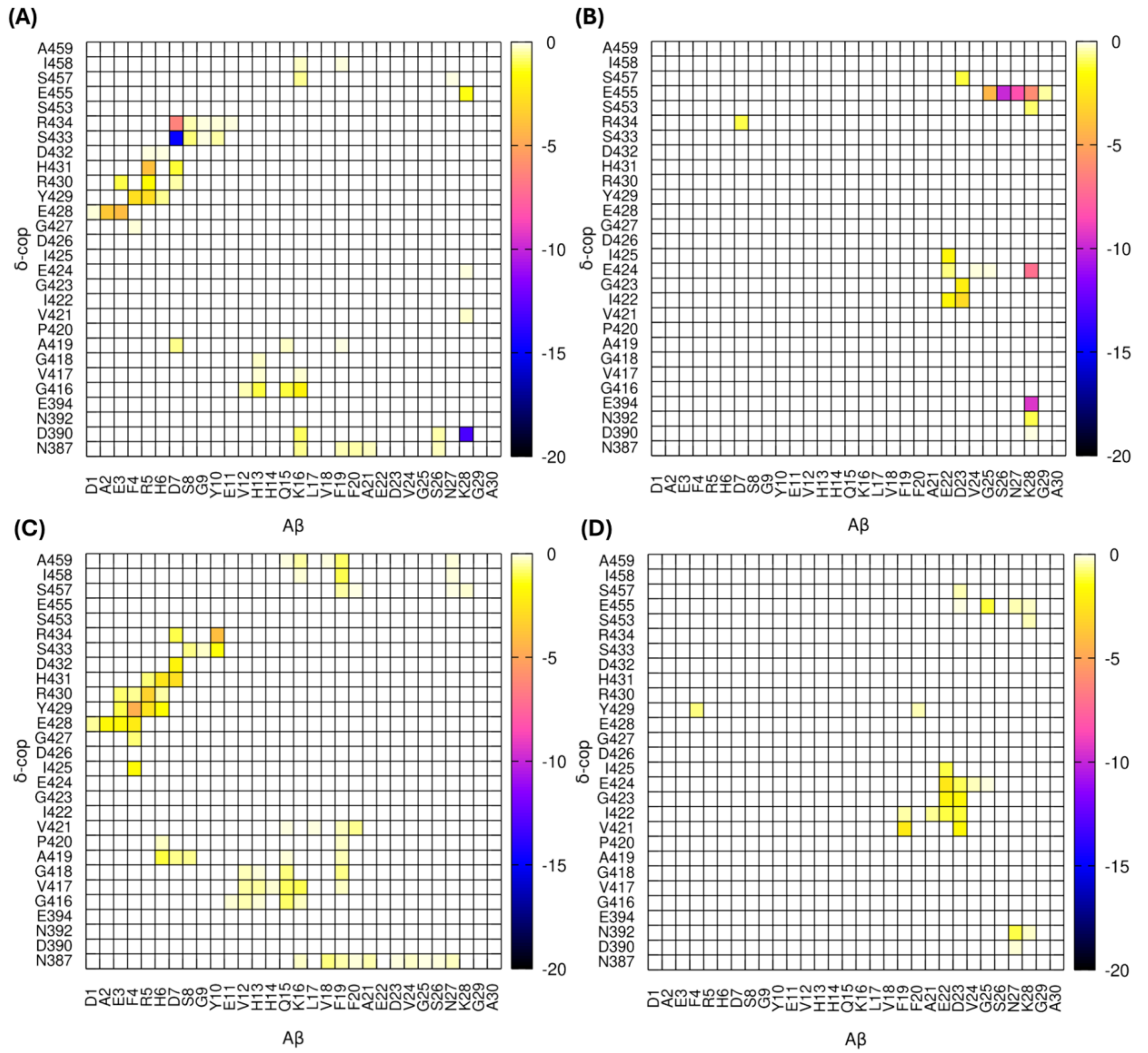

Figure S6. (A,B) Triplicates average of polar interaction free energy maps between residue pairs of δ-COP/Aβ1 monomer and δ-COP/Aβ2 monomer in the lowest binding free energy set of **refined** simulations respectively. **(C,D)** Triplicates average of non-polar interaction free energy maps between residue pairs of δ-COP/Aβ1 monomer and δ-COP/Aβ2 monomer in the lowest binding free energy set of **refined** simulations respectively.

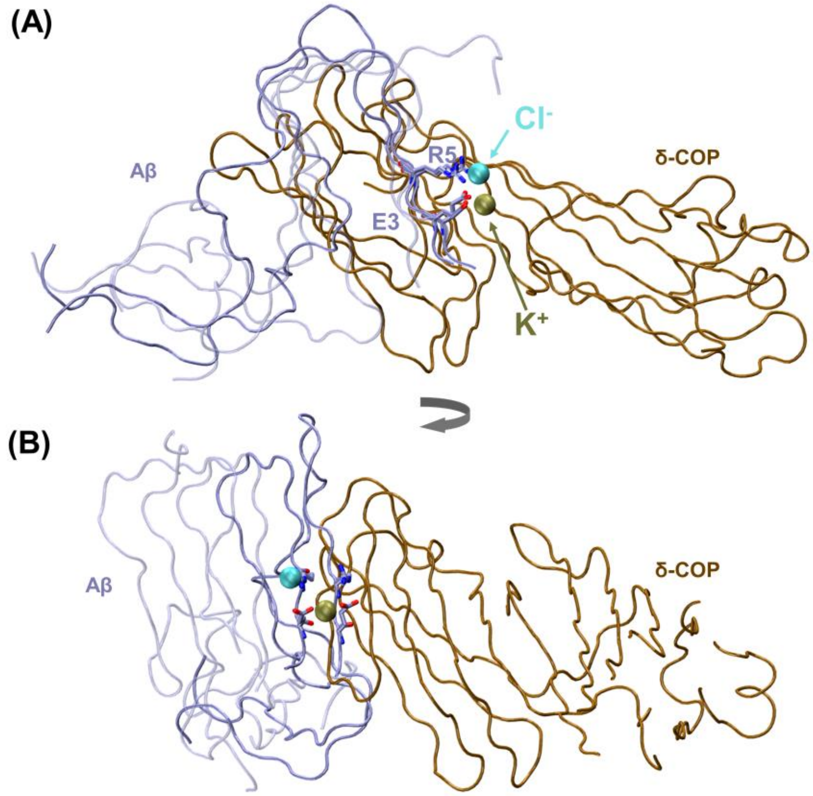

Figure S7. Molecular graphics[5] of δ-COP (ochre tube) complex with Aβ assemblies (ice-blue tube) extracted from the replicate run of the refined simulations with the lowest average binding free energy showing example cases of sodium (tan VdW representation) and chloride ions (cyan VdW representation) in proximity to the negatively charged E3 and positively charged R5 shown with lirorice representation.

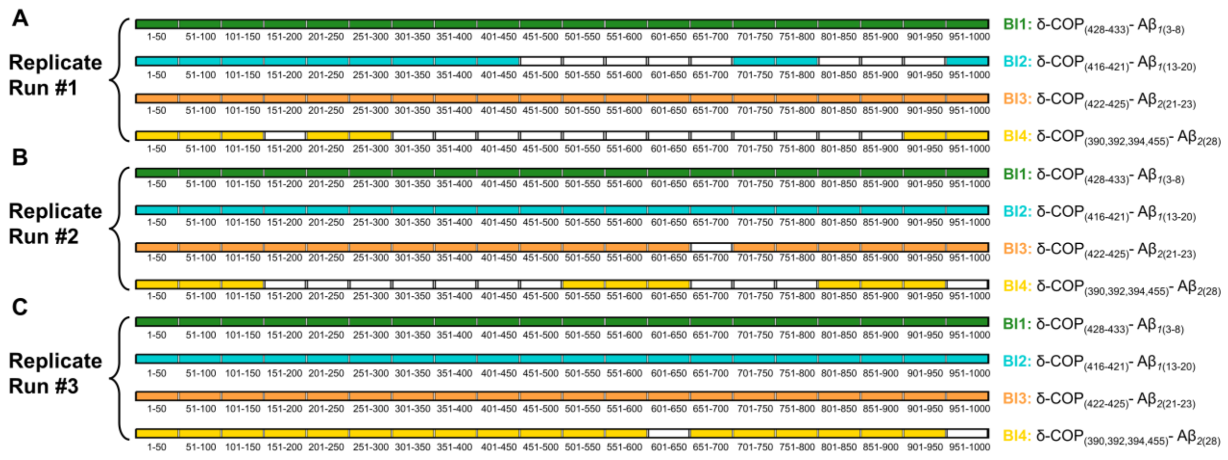

Figure S8. Binary time-window tape-diagrams showing presence (colored) or absence (uncolored) of (i) at least one β-sheet between any residues of δ-COP$_{(428\text{-}433)}$ and Aβ$_{1(3\text{-}8)}$ (green-tape-diagrams), (ii) at least one contact between any residues of δ-COP$_{(416\text{-}425)}$ and Aβ$_{1(13\text{-}23)}$ (cyan-tape-diagrams), (iii) at least one β-sheet between any residues of δ-COP$_{(422\text{-}425)}$ and Aβ$_{2(21\text{-}23)}$ (orange-tape-diagrams), (iv) at least one contact between any residues of δ-COP$_{(390,392,394,455)}$ and Aβ$_{2(28)}$ (yellow-tape-diagrams) **(A)** for replicate run #1, **(B)** for replicate run #2, **(C)** for replicate run # 3 of the lowest binding free energy set of **refined** simulations.

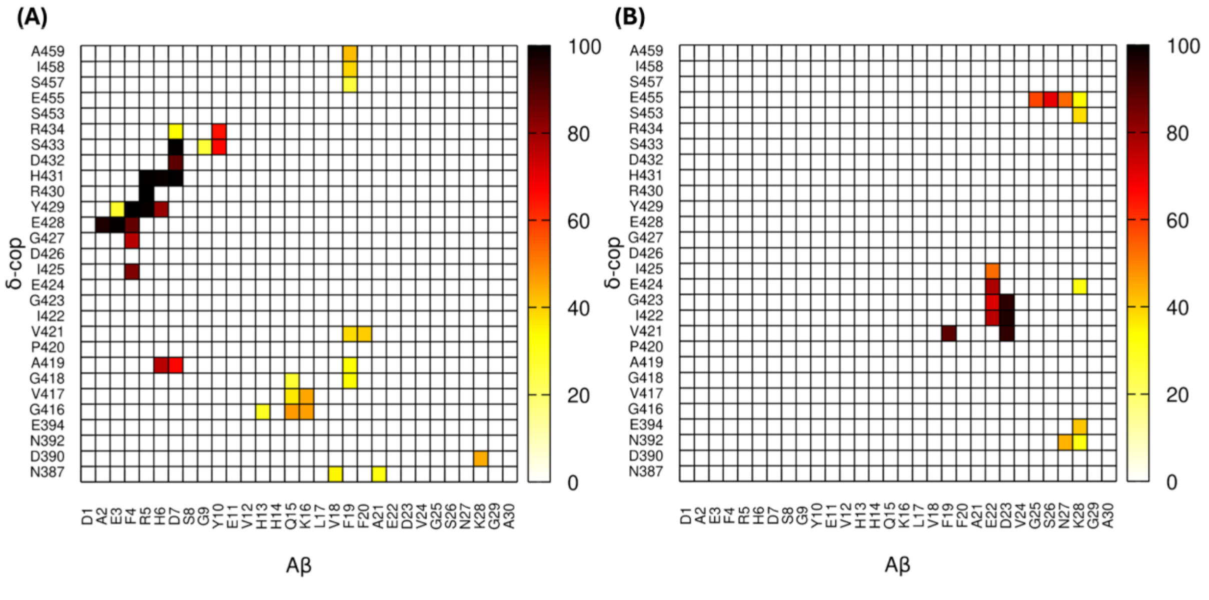

Figure S9. Maps showing the (%) contact probability averaged over triplicate runs, calculated between residue pairs of **(A)** δ-COP and Aβ$_1$ monomer, **(B)** δ-COP and Aβ$_2$ monomer in the lowest binding free energy set of **refined** simulations.

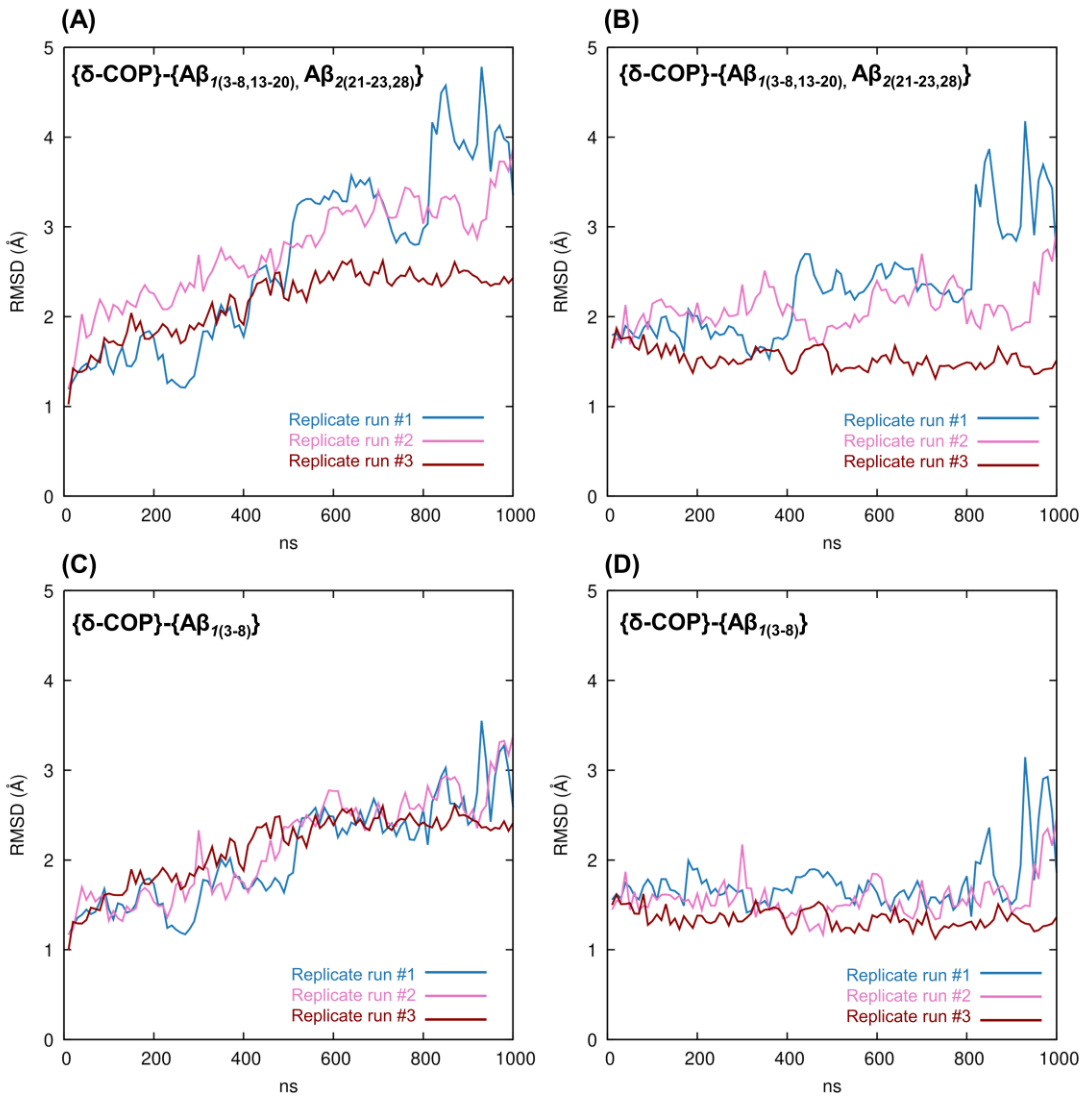

Figure S10. RMSD (Å) of δ-COP and all Aβ residues which belong to all binding interfaces (Aβ$_{1(3-8,13-20)}$, Aβ$_{2(21-23,28)}$) based on **(A)** top and **(B)** average structure. RMSD (Å) of δ-COP and all Aβ residues which belong to *BI1* (Aβ$_{1(3-8)}$) based on **(C)** top and **(D)** average structure. RMSD values represent averages computed over 10-ns time windows.

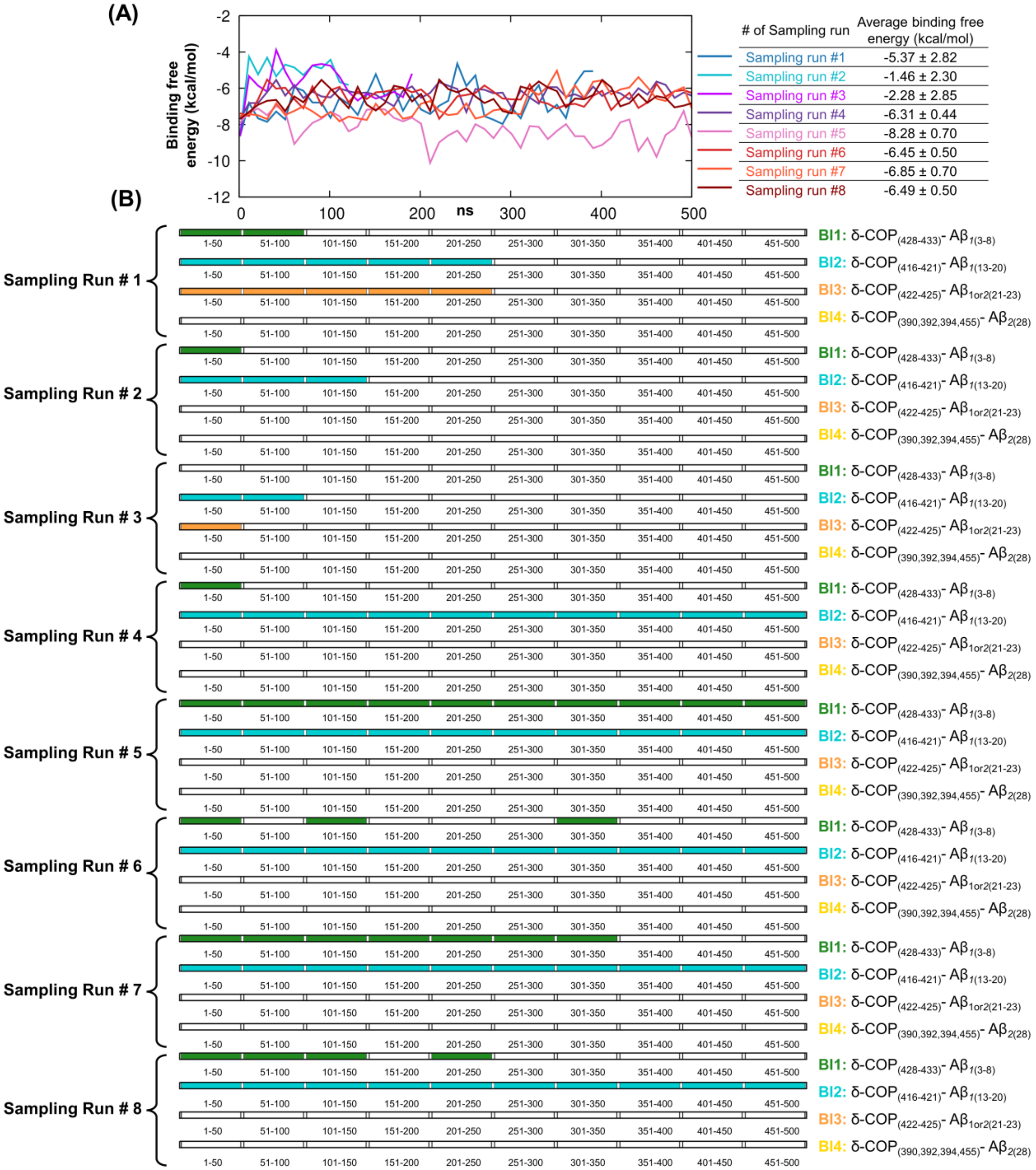

Figure S11. **(A)** Binding free energy (kcal/mol) of δ-COP with Aβ assemblies as a function of the simulated time (ns) for all sampling runs of δ-COP mut complexes with Aβ assemblies, as well as the time average values. **(B)** Binary time-window tape-diagrams showing presence (colored) or absence (uncolored) of (i) at least one β-sheet between any residues of δ-$COP_{(428\text{-}433)}$ and $A\beta_{1(3\text{-}8)}$ (green-tape-diagrams), (ii) at least one contact between any residues of δ-$COP_{(416\text{-}421)}$ and $A\beta_{2(13\text{-}23)}$ (cyan-tape-diagrams), (iii) at least one β-sheet between any residues of δ-$COP_{(422\text{-}425)}$ and $A\beta_{1(21\text{-}23)}$ or $A\beta_{2(21\text{-}23)}$ (orange-tape-diagrams), (iv) at least one contact between any residues of δ-

COP$_{(390,392,394,455)}$ and A$\beta_{2(28)}$ (yellow-tape-diagrams) for all **sampling** runs of δ-COP mut complex with Aβ assemblies.

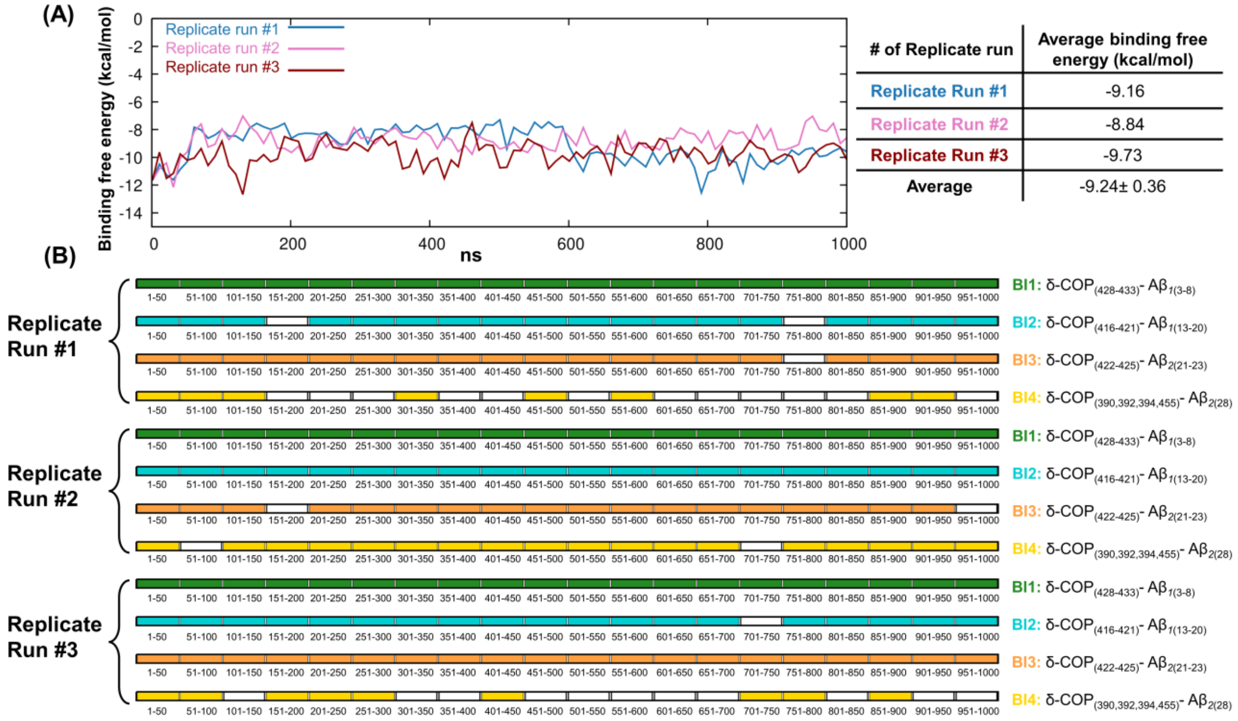

Figure S12. (A) Binding free energy (kcal/mol) of δ-COP with Aβ assemblies as a function of the simulated time (ns) for all refined runs of δ-COP mut complexes with Aβ assemblies, as well as the time average values. **(B)** Binary time-window tape-diagrams showing presence (colored) or absence (uncolored) of (i) at least one β-sheet between any residues of δ-COP$_{(428\text{-}433)}$ and A$\beta_{1(3\text{-}8)}$ (green-tape-diagrams), (ii) at least one contact between any residues of δ-COP$_{(416\text{-}421)}$ and A$\beta_{1(13\text{-}20)}$ (cyan-tape-diagrams), (iii) at least one β-sheet between any residues of δ-COP$_{(422\text{-}425)}$ and A$\beta_{2(21\text{-}23)}$ (orange-tape-diagrams), (iv) at least one contact between any residues of δ-COP$_{(390,392,394,455)}$ and A$\beta_{2(28)}$ (yellow-tape-diagrams) for all **refined** runs of δ-COP mut complex with Aβ assemblies.

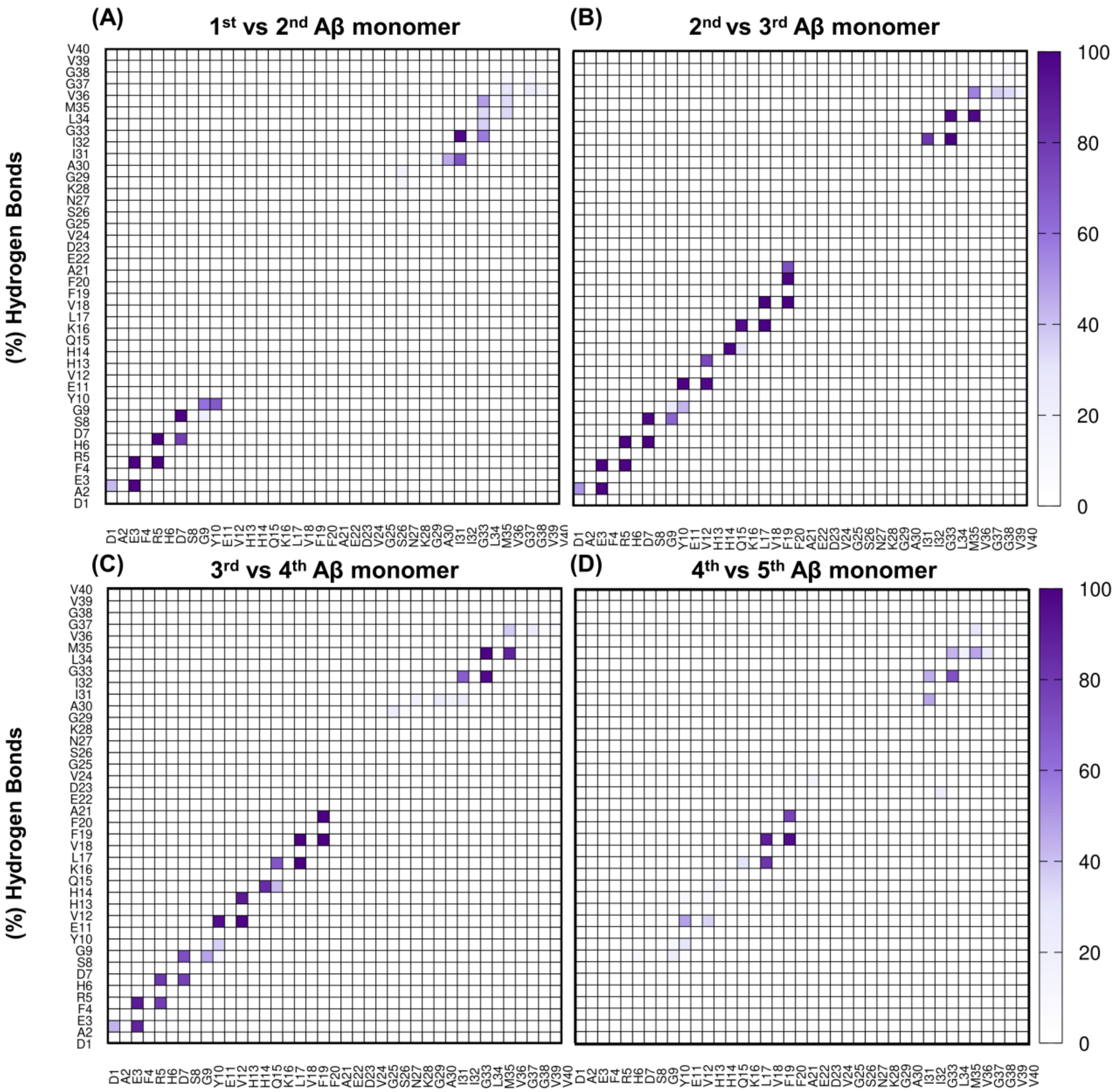

Figure S13. Maps showing the backbone-backbone hydrogen bonds averaged across the triplicate runs of the refined simulations between neighboring Aβ monomers; between **(A)** 1st and 2nd Aβ monomers, **(B)** 2nd and 3rd Aβ monomers, **(C)** 3rd and 4th Aβ monomers, **(D)** 4th and 5th Aβ monomers.

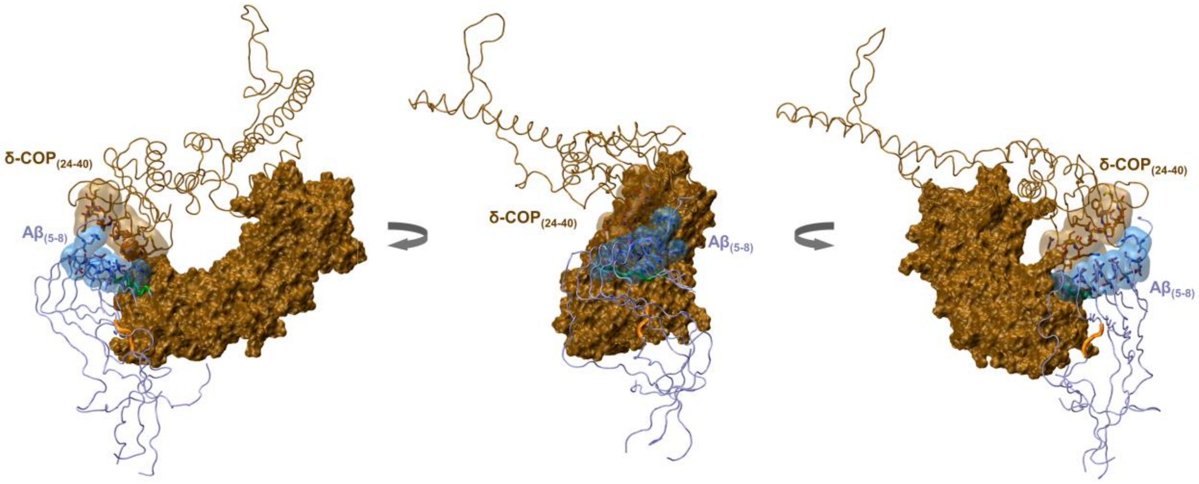

Figure S14. Molecular graphics[5] of the entire δ-COP (1-511) in complex with Aβ assemblies extracted at the last frame (100 ns) of one out of three runs as an indicative case where δ-COP region 24-40 interacts with all amyloid monomers. The interacting interface is shown with licorice and transparent quick surface representation, while δ-COP region 271-511 is shown with surface representation.

References

1 Jumper J, Evans R, Pritzel A, Green T, Figurnov M, Ronneberger O, Tunyasuvunakool K, Bates R, Žídek A, Potapenko A, Bridgland A, Meyer C, Kohl SAA, Ballard AJ, Cowie A, Romera-Paredes B, Nikolov S, Jain R, Adler J, Back T, Petersen S, Reiman D, Clancy E, Zielinski M, Steinegger M, Pacholska M, Berghammer T, Bodenstein S, Silver D, Vinyals O, Senior AW, Kavukcuoglu K, Kohli P, Hassabis D. Highly accurate protein structure prediction with AlphaFold. Nature. 2021;596(7873):583-589.

2 Varadi M, Bertoni D, Magana P, Paramval U, Pidruchna I, Radhakrishnan M, Tsenkov M, Nair S, Mirdita M, Yeo J, Kovalevskiy O, Tunyasuvunakool K, Laydon A, Žídek A, Tomlinson H, Hariharan D, Abrahamson J, Green T, Jumper J, Birney E, Steinegger M, Hassabis D, Velankar S. AlphaFold Protein Structure Database in 2024: providing structure coverage for over 214 million protein sequences. Nucleic Acids Res. 2024;52(D1):D368-D375.

3 Varadi M, Anyango S, Deshpande M, Nair S, Natassia C, Yordanova G, Yuan D, Stroe O, Wood G, Laydon A, Žídek A, Green T, Tunyasuvunakool K, Petersen S, Jumper J, Clancy E, Green R, Vora A, Lutfi M, Figurnov M, Cowie A, Hobbs N, Kohli P, Kleywegt G, Birney E, Hassabis D, Velankar S. AlphaFold Protein Structure Database: massively expanding the structural coverage of protein-sequence space with high-accuracy models. Nucleic Acids Res. 2022;50(D1):D439-D444.

4 Kollmer M, Close W, Funk L, Rasmussen J, Bsoul A, Schierhorn A, Schmidt M, Sigurdson CJ, Jucker M, Fändrich M. Cryo-EM structure and polymorphism of Aβ amyloid fibrils purified from Alzheimer's brain tissue. Nat Commun. 2019;10(1):4760.

5 Humphrey W, Dalke A, Schulten K. VMD: visual molecular dynamics. J Mol Graph. 1996;14(1):33-8, 27-8.

6 Jo S, Kim T, Iyer VG, Im W. CHARMM-GUI: a web-based graphical user interface for CHARMM. J Comput Chem. 2008;29(11):1859-65.

7 Brooks BR, Brooks CL 3rd, Mackerell AD Jr, Nilsson L, Petrella RJ, Roux B, Won Y, Archontis G, Bartels C, Boresch S, Caflisch A, Caves L, Cui Q, Dinner AR, Feig M, Fischer S, Gao J, Hodoscek M, Im W, Kuczera K, Lazaridis T, Ma J, Ovchinnikov V, Paci E, Pastor RW, Post CB, Pu JZ, Schaefer M, Tidor B, Venable RM, Woodcock HL, Wu X, Yang W, York DM, Karplus M. CHARMM: the biomolecular simulation program. J Comput Chem. 2009;30(10):1545-614.

8 Lee J, Cheng X, Swails JM, Yeom MS, Eastman PK, Lemkul JA, Wei S, Buckner J, Jeong JC, Qi Y, Jo S, Pande VS, Case DA, Brooks CL 3rd, MacKerell AD Jr, Klauda JB, Im W. CHARMM-GUI Input Generator for NAMD, GROMACS, AMBER, OpenMM, and CHARMM/OpenMM Simulations Using the CHARMM36 Additive Force Field. J Chem Theory Comput. 2016;12(1):405-13.

9 Eastman P, Swails J, Chodera JD, McGibbon RT, Zhao Y, Beauchamp KA, Wang LP, Simmonett AC, Harrigan MP, Stern CD, Wiewiora RP, Brooks BR, Pande VS. OpenMM 7: Rapid development of high performance algorithms for molecular dynamics. PLoS Comput Biol. 2017;13(7):e1005659.

10 Xue LC, Rodrigues JP, Kastritis PL, Bonvin AM, Vangone A. PRODIGY: a web server for predicting the binding affinity of protein-protein complexes. Bioinformatics. 2016;32(23):3676-3678.

11 Vangone A, Bonvin AM. Contacts-based prediction of binding affinity in protein-protein complexes. Elife. 2015;4:e07454.

12 Orr AA, Wördehoff MM, Hoyer W, Tamamis P. Uncovering the Binding and Specificity of β Wrapins for Amyloid-β and α-Synuclein. J Phys Chem B. 2016;120(50):12781-12794.

13 Orr AA, Shaykhalishahi H, Mirecka EA, Jonnalagadda SVR, Hoyer W, Tamamis P. Elucidating the multi-targeted anti-amyloid activity and enhanced islet amyloid polypeptide binding of β-wrapins. Comput Chem Eng. 2018 ;116:322-332.

14 Orr AA, Gonzalez-Rivera JC, Wilson M, Bhikha PR, Wang D, Contreras LM, Tamamis P. A high throughput and rapid computational method for screening of RNA post-transcriptional modifications that can be recognized by target proteins. Methods. 2018;143:34-47.

15 Gonzalez-Rivera JC, Orr AA, Engels SM, Jakubowski JM, Sherman MW, O'Connor KN, Matteson T, Woodcock BC, Contreras LM, Tamamis P. Computational evolution of an RNA-binding protein towards enhanced oxidized-RNA binding. Comput Struct Biotechnol J. 2019;18:137-152.

16 Orr AA, Kuhlmann SK, Tamamis P. Computational design of a β-wrapin's N-terminal domain with canonical and non-canonical amino acid modifications mimicking curcumin's proposed inhibitory function. Biophys Chem. 2022;286:106805

17 Miller LG, Kim W, Schowe S, Taylor K, Han R, Jain V, Park R, Sherman M, Fang J, Ramirez H, Ellington A, Tamamis P, Resendiz MJE, Zhang YJ, Contreras L. Selective 8-oxo-rG stalling occurs in the catalytic core of polynucleotide phosphorylase (PNPase) during degradation. Proc Natl Acad Sci U S A. 2024;121(46):e2317865121.

18 Lee MS, Salsbury Jr FR, Brooks III CL. Novel generalized Born methods. The Journal of chemical physics. 2002;116(24):10606-14.

19 Lee MS, Feig M, Salsbury Jr FR, Brooks III CL. New analytic approximation to the standard molecular volume definition and its application to generalized Born calculations. Journal of computational chemistry. 2003;24(11):1348-56.

20 Wang KW, Lee J, Zhang H, Suh D, Im W. CHARMM-GUI Implicit Solvent Modeler for Various Generalized Born Models in Different Simulation Programs. J Phys Chem B. 2022;126(38):7354-7364.
